# The development of audio–visual temporal precision precedes its rapid recalibration

**DOI:** 10.1038/s41598-022-25392-y

**Published:** 2022-12-14

**Authors:** Shui’er Han, Yi-Chuan Chen, Daphne Maurer, David I. Shore, Terri L. Lewis, Brendan M. Stanley, David Alais

**Affiliations:** 1 Department of Brain and Cognitive Sciences, University of Rochester, Rochester, NY USA; 2 Institute for Infocomm Research, Agency for Science, Technology and Research, Singapore, Singapore; 3 Department of Medicine, Mackay Medical College, New Taipei City, Taiwan; 4 Department of Psychology, Neuroscience and Behaviour, McMaster University, Hamilton, Canada; 5 The Multisensory Mind Inc., Hamilton, ON Canada; 6 School of Psychology, The University of Sydney, Sydney, Australia

**Keywords:** Perception, Human behaviour

## Abstract

Through development, multisensory systems reach a balance between stability and flexibility: the systems integrate optimally cross-modal signals from the same events, while remaining adaptive to environmental changes. Is continuous intersensory recalibration required to shape optimal integration mechanisms, or does multisensory integration develop prior to recalibration? Here, we examined the development of multisensory integration and rapid recalibration in the temporal domain by re-analyzing published datasets for audio–visual, audio–tactile, and visual–tactile combinations. Results showed that children reach an adult level of precision in audio–visual simultaneity perception and show the first sign of rapid recalibration at 9 years of age. In contrast, there was very weak rapid recalibration for other cross-modal combinations at all ages, even when adult levels of temporal precision had developed. Thus, the development of audio–visual rapid recalibration appears to require the maturation of temporal precision. It may serve to accommodate distance-dependent travel time differences between light and sound.

## Introduction

Neural plasticity enables the human brain to adapt to the environment throughout the lifespan. From birth, we learn to associate sensory signals from different modalities when they arise from the same event in the external world^1^. Because sensory systems develop at different rates, some have suggested that multisensory coordination and integration build on intersensory calibration and continuous recalibration during development^2,3^. Presumably, the intersensory recalibrations should be extensive at younger ages and then decrease as maturation progresses, because with age, the systems become more specialized to native stimuli and neural plasticity decreases. Recently, however, Rohlf, Li, Bruns, and Röder^4^ demonstrated a reversed trend by showing that audio–visual integration in the spatial domain develops prior to the emergence of spatial recalibration to newly experienced spatial disparity. Here we examine this surprising developmental trend in the *temporal* domain among three sensory combinations—audio–visual, audio–tactile, and visual–tactile—by re-analyzing previously published data^5–7^.

The ability to integrate cross-modal signals supports our survival in a multisensory world: multisensory integration improves precision, accuracy, and processing speed of perception^8–13^. Temporal simultaneity provides one crucial cue in determining which cross-modal signals belong together^14,15^, while those integration benefits tend to decline when temporal asynchronies are introduced between the component signals^16,17^. In real world contexts, the relative timing of signals generated by an external event can vary significantly between sensory modalities. Specifically, the timing of audio–visual signals is dependent on source distance in audition but not in vision because sound travels much slower than light; on the other hand, a tactile stimulus is always applied to the body surface (i.e., directly to the sensory system). Once at the sensory periphery, there are internal factors such as differences in neural transduction times^18^: visual processing is the slowest^19^, and tactile processing time is positively correlated with the neural transduction distance^20^. Temporal asynchrony is therefore inevitable between stimulus signals arising from a common event.

To deal with the temporal variations of multisensory signals that originate from the same event, the multisensory system realigns cross-modal signals according to previous experience, a phenomenon known as temporal recalibration (see^15^, for a review). Temporal recalibration is manifested by shifts in the observer’s point of subjective simultaneity (PSS)—the time lag at which sensory signals are perceived as most likely to be simultaneous. For example, adapting to a visual-leading pair biases the PSS toward visual-leading presentations^21–29^. There are two forms of temporal recalibration at different time scales. Slow recalibration involves an exposure phase during which observers are presented repeatedly with a fixed time lag between signals (e.g., an auditory signal constantly lags a visual signal by 200 ms) for a period of time (e.g., several minutes) before demonstrating a change in tested simultaneity perception^21,22^. Following several minutes of adaptation, the recalibration effect lasts for about a minute^23,24^. Rapid recalibration, on the other hand, involves a change after a time lag on the *single* previous trial, but is influenced far less by the time lag of one further trial back^25^. Hence, slow and rapid recalibrations demonstrate quick adaptations and recoveries from stimulus variations of multisensory systems—such dynamic *malleability* is separate from *plasticity* which often involves experience-dependent changing of neural substrates. In adults, both rapid and slow recalibrations are more reliable for audio–visual than in audio–tactile or visual–tactile pairings^23,30,31^.

From a developmental perspective, the capability to detect temporal proximity between multisensory stimuli is observed at birth or even earlier in the fetal stage^32–34^. Nevertheless, precision of multisensory simultaneity perception is relatively low at these earlier stages, and its developmental trajectory is protracted and varies across sensory pairings (see Fig. 3 in^7^). Precision improves with age and reaches adult levels by 9 years for audio–visual pairings and by 11 years for visual–tactile and audio–tactile pairings. The PSS, however, is adult-like by the youngest ages tested (5 years in audio–visual pairings, and 7 years in visual–tactile and audio–tactile pairings^5–7^).

Given the early maturation of the PSS, it remains an open question whether young children exhibit temporal recalibration, and if so, how it might vary through development and for different sensory combinations. The sensitivity to temporal correspondence at very early ages and the early maturation of the PSS suggest that temporal recalibration may be present early in life*.* However*,* in practice, testing temporal recalibration would be an enormous procedural challenge with children, especially the long adaptation periods for the slow recalibration. On the other hand, the development of temporal processing throughout childhood suggests that rapid temporal recalibration may be present and linked to the maturation of simultaneity perception^35^ (see^4,36^ in the spatial domain). Rapid recalibration is easily uncovered by a sequential analysis of the data of simultaneity judgments^25,35^.

**Figure 1:**
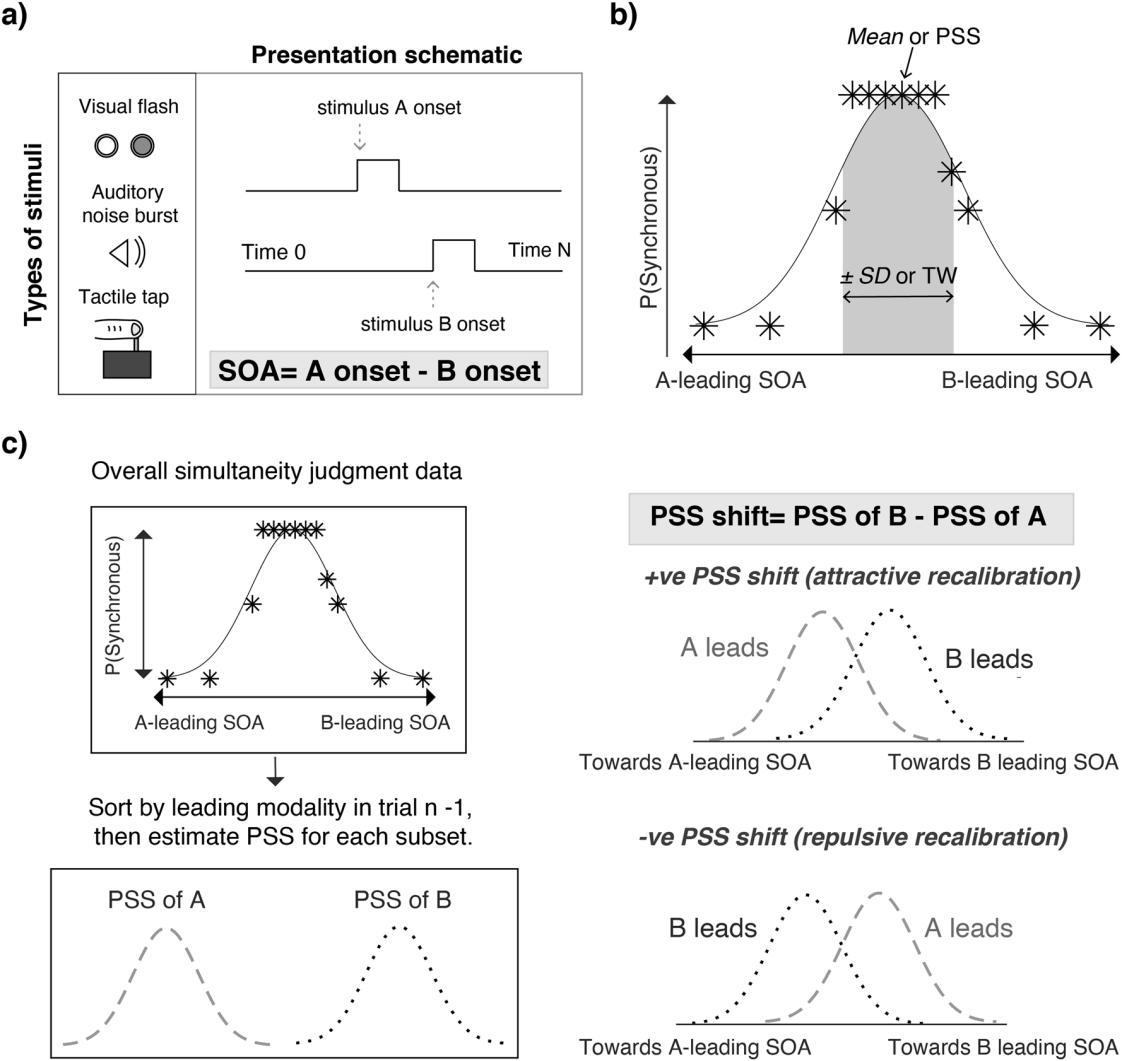
Obtaining the parameters of simultaneity perception. (**a**) Cross-modal stimuli were a visual flash, auditory noise burst, or a tactile tap on the finger. These were presented at 13 levels of stimulus onset asynchrony (SOA), and each participant reported orally whether they perceived the cross-modal pair to be synchronous. (**b**) The simultaneity judgment data (denoted by asterisks) collected from (**a**) were then fitted with a Gaussian function, where the amplitude, standard deviation (*SD*), and mean were free parameters. The mean and standard deviation were used to represent the point of subjective simultaneity (PSS) and the width of temporal simultaneity window, respectively. (**c**) Computation of PSS shift. To estimate the effect of rapid temporal recalibration, the simultaneity judgment data (denoted by asterisks) was first sorted by the leading modality in the previous trial. This produced two subsets of data, one preceded by a modality A-leading trial and one preceded by a modality B-leading trial. The subsets were then fitted with Gaussian functions, which provided the PSS of each subset. Using the respective PSS estimates, we computed the PSS shift by subtracting PSS_A_ from PSS_B._

The current study examined the developmental trajectories of rapid temporal recalibration for three cross-modal pairs (i.e., audio–visual, audio–tactile, and visual–tactile; Fig. 1a), and their respective relations with age-related changes in the precision of simultaneity perception. To do so, we re-analyzed the datasets of three previous developmental studies^5–7^ that measured simultaneity perception in children and compared those data to adult groups. If cross-sensory calibration serves as the basis for the development of multisensory integration^2^, then we would expect rapid recalibration to emerge early and then decrease as multisensory simultaneity perception matures^35^. Alternatively, rapid recalibration might develop after the system matures, providing a form of malleability to accommodate the temporal variations among multisensory events^4^.

## Results

Two main measures were computed in our re-analysis, namely, the width of the temporal simultaneity window and the PSS shifts associated with rapid recalibration. Briefly, the width of the temporal simultaneity window of each participant was estimated using Gaussian distribution fits on the respective cross-modal pairing (Fig. 1b). To compute the magnitude of rapid temporal recalibration, each participant’s data for each cross-modal combination was first sorted into two bins based on the leading modality in the preceding trial. Gaussian distributions were then fitted to these subsets and the difference in distribution means yielded the PSS shift for the cross-modal combination (Fig. 1c). Further details about the analyses are provided in the Methods section.

### Rapid recalibration develops with age for audio–visual pairings

**Figure 2:**
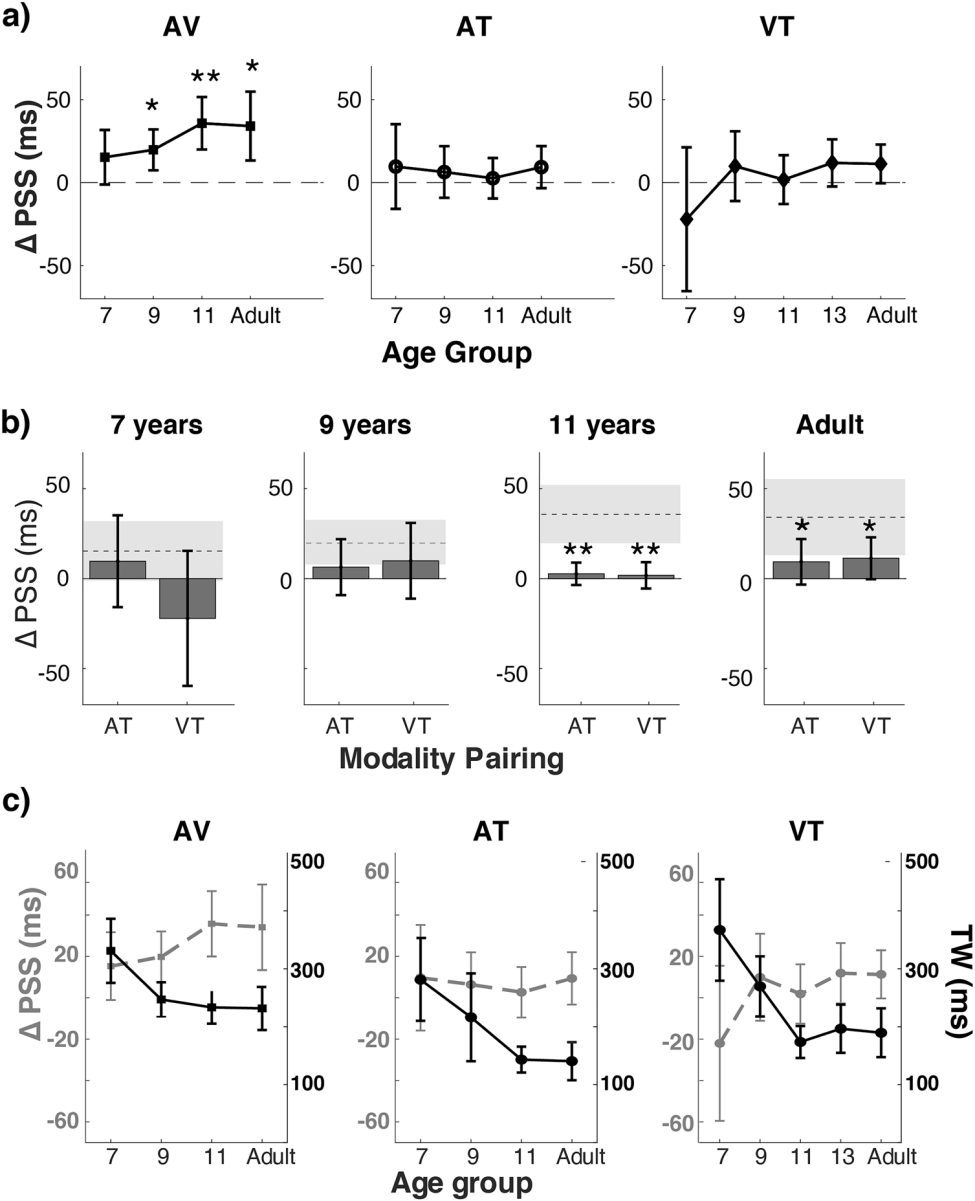
Effect of age on rapid temporal recalibration and the width of the simultaneity window (**a**) The developmental trajectories of PSS shifts (representing the size of rapid recalibration) are plotted for audio–visual (AV; left panel), audio–tactile (AT; middle panel), and visual–tactile (VT; right panel) pairings. Compared to a hypothesized PSS shift of 0 ms, PSS shifts for audio–visual pairings were statistically significant at 9 years and older ages. In contrast, none of the age groups showed a significant shift for the audio–tactile and visual–tactile pairings. (**b**) PSS shifts were larger for audio–visual pairings than for the other two cross-modal pairings by 11 years of age and in adults. Dashed lines represent the average PSS shifts for audio–visual pairing and the grey shaded areas represent 95% confidence intervals. (**c**) The developmental trajectories of the width of the simultaneity window. Compared to the trends in PSS shifts (superimposed, grey dashed lines), the width of temporal window decreased with age for all cross-modal pairings. All error bars represent 95% confidence intervals. Asterisks represent significance on at least one of the statistical tests (i.e., one-sample t-tests or bootstrap hypothesis tests; *smallest *p* < .05, **smallest *p* < .01).

When each age group was compared to a hypothesized PSS shift of 0 ms (see Fig. 2a), audio–visual temporal recalibration was significant for 9 years of age or older (9-year-olds: *t*(18) = 3.15, *p* = 0.02, *d* = 0.72, bootstrapped *p* < 0.001; 11-year-olds: *t*(19) = 4.44, *p* = 0.001, *d* = 0.99, bootstrapped *p* < 0.001; adults: (*t*(19) = 3.23, *p* = 0.02, *d* = 0.72, bootstrapped *p* = 0.004), but not at 7 years of age (*t*(18) = 1.83, *p* = 0.08, *d* = 0.42, bootstrapped *p* = 0.053). For the audio–tactile or visual–tactile pairings, none of the age groups differed significantly from 0 ms (all *p*s > 0.05 for one-sample *t*-tests and bootstrap hypothesis tests), suggesting that rapid temporal recalibration processes remain weak for audio–tactile and visual–tactile pairings throughout development.

### Post-maturation audio–visual PSS shifts are larger than other combinations

Figure 2B compares the PSS shifts in audio–tactile and visual–tactile pairings against audio–visual pairings recorded for 7-, 9-, 11-year-olds, and adults. For the youngest age group, audio–visual PSS shifts were statistically comparable to those obtained with audio–tactile pairings (*t*(30) = 0.38, *p* = 0.71, *d* = 0.14, bootstrapped *p* = 0.65) and visual–tactile pairings (*t*(33) = 1.89, *p* = 0.07, *d* = 0.64, bootstrapped *p* = 0.05). Similarly, in the 9-year-olds, audio–visual PSS shifts did not differ significantly from audio-tactile (*t*(36) = 1.32, *p* = 0.19, *d* = 0.42, bootstrapped *p* = 0.18) and visual–tactile (*t*(36) = 0.79, *p* = 0.43, *d* = 0.25, bootstrapped *p* = 0.44) pairings. However, by 11 years of age, PSS shifts were significantly larger in audio–visual conditions than in audio–tactile (*t*(38) = 3.25, *p* = 0.002, *d* = 1.06, bootstrapped *p* < 0.001) and visual–tactile (*t*(38) = 3.12, *p* = 0.003, *d* = 0.99, bootstrapped *p* = 0.001) conditions. The larger PSS shifts in audio–visual than audio–tactile (*t*(38) = 2.00, *p* = 0.05, *d* = 0.63, bootstrapped *p* = 0.03) and visual–tactile (*t*(38) = 1.88, *p* = 0.07, *d* = 0.59, bootstrapped *p* = 0.04) pairings continued in adulthood.

### Post-maturation temporal precision correlates with audio–visual PSS shifts

The effect of age on the width of the simultaneity window is contrasted with its effect on the PSS shift in Fig. 2c. Overall, the width of the simultaneity window decreased with age for audio–visual pairings (*F*(3,74) = 5.63, *p* = 0.002, *η*_*p*_^2^ = 0.19, bootstrapped *p* < 0.001), audio–tactile pairings (*F*(3,68) = 5.54, *p* = 0.002, *η*_*p*_^2^ = 0.20, bootstrapped *p* = 0.001), and visual–tactile conditions (*F*(4,89) = 8.83, *p* < 0.001, *η*_*p*_^2^ = 0.28, bootstrapped *p* < 0.001). Post-hoc pairwise comparisons showed that for audio–visual and audio–tactile presentations, main effects were driven by the 7-year-old groups, which had wider windows than the older age groups (*ps* < 0.05). Similarly, for visual–tactile pairings, 7- and 9-year-olds had larger window sizes than the other ages (*p*s < 0.05). In contrast, age did not have a significant effect on PSS shifts for the audio–visual pairing (*F*(3,74) = 1.44, *p* = 0.24, *η*_*p*_^2^ = 0.06, bootstrapped *p* = 0.06), the audio–tactile pairing (*F*(3,68) = 0.17, *p* = 0.92, *η*_*p*_^2^ = 0.01, bootstrapped *p* = 0.49), and the visual–tactile pairing (*F*(4,89) = 1.71, *p* = 0.15, *η*_*p*_^2^ = 0.07, bootstrapped *p* = 0.20).

**Figure 3:**
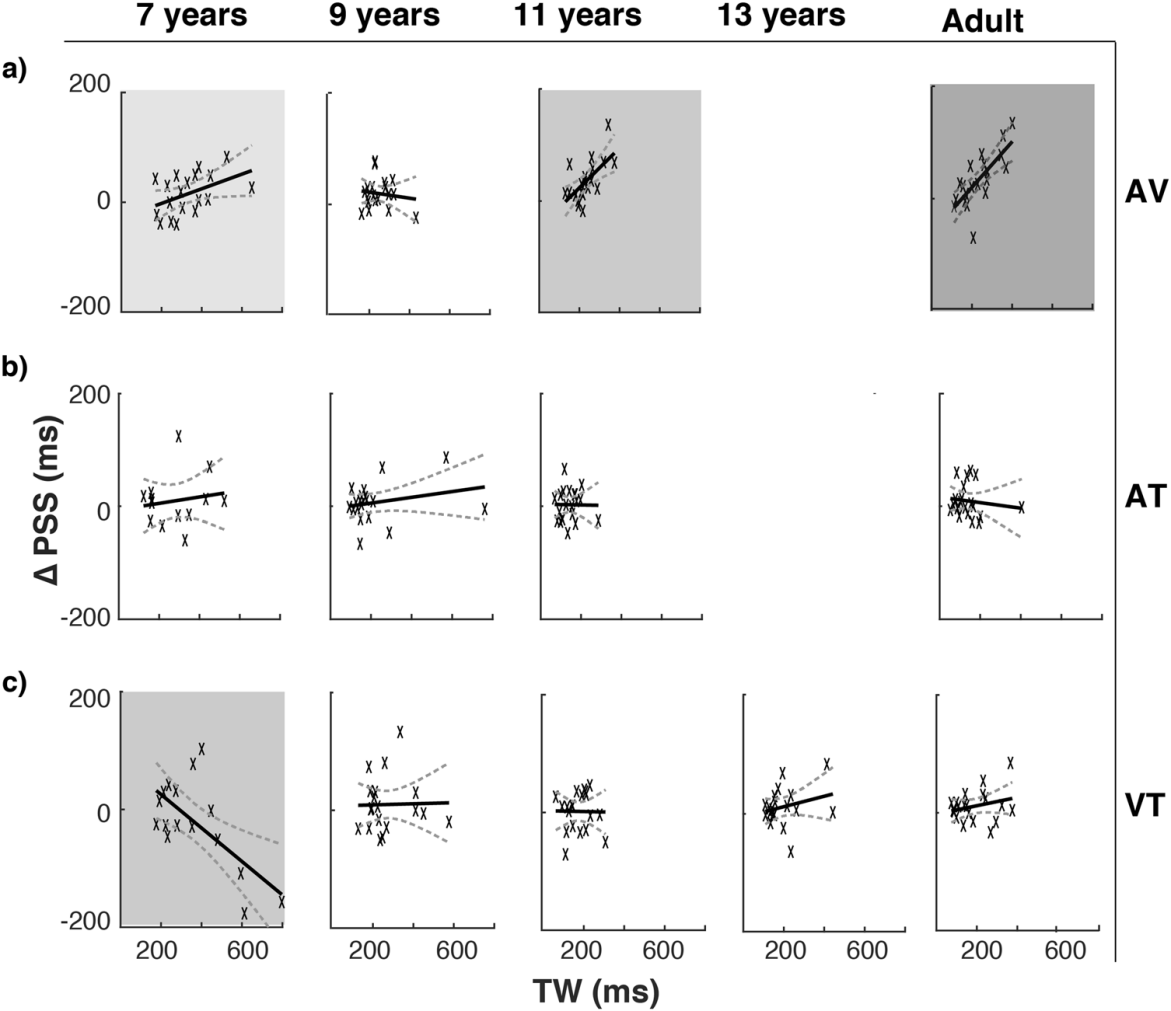
The relation between PSS shifts and the widths of the temporal window of simultaneity for (**a**) audio–visual, (**b**) audio–tactile and (**c**) visual-tactile pairings. Wider audio–visual windows are typically accompanied by larger PSS shifts^25^, but this relation becomes reliably significant only at 11 years of age and adults. Audio-tactile pairings do not follow a similar relation, demonstrating only a reduced variance from 9 years of age onwards. Similarly, visual–tactile pairings showed a reduced variance from 9 years of age onwards. At 7 years of age (shaded bottom–left panel), visual–tactile simultaneity judgments demonstrated a negative correlation, which was found to be statistically significant. Shaded areas represent significance on at least one of the statistical tests (i.e., linear regression analyses and/or bootstrap hypothesis tests; lightest grey: smallest *p* < .05, mid grey: smallest *p* < .01, darkest grey: smallest *p* < .001).

Further within-age-group analyses showed that the correlation between the PSS shifts and the widths of the simultaneity window was dependent on age and the modality combination. Specifically, positive correlations between larger PSS shifts and wider audio–visual simultaneity windows were demonstrated reliably by 11 years of age (Fig. 3a; 11-year-olds: *ß* = 0.36, *t*(18) = 3.47, *p* = 0.003, bootstrapped *p* = 0.009; adult: *ß* = 0.34, *t*(18) = 5.29, *p* < 0.001, bootstrapped *p* < 0.001) but not reliable in the younger age groups (7-year-olds: *ß* = 0.13, *t*(17) = 2.07, *p* = 0.05, bootstrapped *p* = 0.02; 9-year-olds: *ß* = −0.06, *t*(17) = -0.55, *p* = 0.59, bootstrapped *p* = 0.71). The other cross-modal pairings did not demonstrate a convincing relation between the PSS shift and the width of the simultaneity window (Table 1), showing instead a decrease in response variability with age (Fig. 3b–c). The only exception was the 7-year-olds in the visual–tactile condition (Fig. 3c, bottom-left panel), where an inverse correlation was observed between the PSS shift and the width of the simultaneity window (*ß* = -0.28, *t*(14) = 3.33, *p* = 0.005, bootstrapped *p* = 0.04).

**Table 1:** Results of the regression analyses between the PSS shifts and the widths of simultaneity window for the three cross-modal pairings across age groups.

Condition		Age group	Slope	*R*^2^_*adj*_	*p*	Bootstrapped* p*
Audio–visual		7-year-olds	0.13	0.16	.05	.02*
		9-year-olds	−0.06	−0.04	.59	.71
		11-year-olds	0.36	0.37	.003*	.009*
		13-year-olds	–	–	–	–
		Adults	0.41	0.52	.0002*	< .0001*
Audio–tactile		7-year-olds	0.06	−0.06	.61	.39
		9-year-olds	0.05	0.01	.30	.51
		11-year-olds	−0.01	−0.06	.96	.98
		13-year-olds	–	–	–	–
		Adults	−0.05	−0.04	.59	.34
Visual–tactile		7-year-olds	−0.28	0.40	.005*	.04*
		9-year-olds	0.01	−0.06	.92	.87
		11-year-olds	−0.01	−0.05	.95	.99
		13-year-olds	0.09	0.01	.29	.48
		Adults	0.07	0.01	.28	.41

Asterisks indicate that the slope of the statistical tests was significant (i.e., linear regression analyses or bootstrap hypothesis tests).

## Discussion

We investigated the development of rapid recalibration in the temporal domain and its relation with the simultaneity window across three cross-modal pairings. The development of rapid recalibration was protracted and unique to audio–visual presentations: by 9 years of age, a significant shift of PSS attributed to rapid recalibration was observed (Fig. 2a, left panel). In contrast, none of the age groups demonstrated such PSS shifts for auditory–tactile or visual–tactile pairings (Fig. 2a, centre and right panels). By 11 years of age, PSS shifts for the audio–visual pairing were reliably larger than those for the other two cross-modal pairings, and a positive correlation between PSS shifts and the widths of the simultaneity window for the audio–visual pairing was observed reliably.

For the audio–visual pairing, rapid recalibration first appeared at age 9 and continued with age; in contrast, children’s precision of simultaneity perception (i.e., the width of the temporal window) improved with age up to age 9, at which point it was adultlike^5^. Combined, these results suggest that children can realign the temporal synchrony of recent audio–visual events rapidly *only after* audio–visual simultaneity perception matures, and not before. This result contrasts with that of Noel et al.^35^ who demonstrated that rapid recalibration peaked at 12.1 years of age and then decreased, while the precision of audio–visual simultaneity perception increased until late adolescence (17–18 years of age), suggesting a developmental trend of the audio–visual system from malleable to stable. However, Noel et al. had only nine participants in the age range of 7 to 10 years—the critical age range according to our data—and used a broad sliding window that averaged across 7–11 years of age. Doing so would miss the critical changes we observed with 20 children per age group for ages 7-, 9-, and 11-year-olds (see Footnote 1 in^7^).

The earlier development of the precision of audio–visual perception than rapid recalibration in both the temporal and spatial domains (the current study and^4^) suggests that plasticity/malleability of the audio–visual system does not decrease monotonically with age. Children’s wider simultaneity window^5^ and larger spatial ventriloquism effect^4^ indicate that they tend to integrate visual and auditory information originating from disparate timings or locations more often, and less selectively, than do adults. During development, the audio–visual system is plastic in order to accommodate physical growth of the body and the maturation of each sensory system. In addition, intersensory calibration based on daily audio–visual experience will lead to changes based on the more accurate (i.e., the less noisy) modality, the precision of each sensory system, and expectations about signals that ought to be integrated (i.e., the prior of common cause^37,38^). In turn, the audio–visual system develops a statistically optimal algorithm for signal integration in both spatial and temporal domains that is useful for most daily events^8,39–41^. During this developmental period, accommodating to particular audio–visual events which may occur occasionally or exceptionally (such as the rapid recalibration to the most recent event) might lead to a misrepresentation of the optimal window or even a prolonged developmental trajectory if the window keeps changing.

After the audio–visual system achieves optimal precision for integration, it remains malleable in both the temporal and spatial domains, even in adulthood. This is demonstrated in the phenomena of rapid recalibration^25,42^, slow recalibration^21,22,43^, and perceptual training with feedback^43–45^. Hence, the plasticity/malleability of the audio–visual system tends to pass through two developmental stages during which the system is susceptible to multisensory events at distinct time scales (see^1^). The first is a long-term scale beginning at birth and lasting until late childhood during which the multisensory system is tuned by general events from an overly broad system into optimal precision. This development of optimal integration is based on continuously improving sensory reliability and establishing the prior of common cause for multisensory signals^4,46–48^ (see^49,50^ for other cross-modal combinations). Afterward, when the system is relatively stable, the second time scale takes effect with mild short-term influences from recent experience. The rapid recalibration underpinned by short-term malleability may rely mainly on accommodating the prior of common cause and not depend as much on signal reliability^51–53^. For example, rapid recalibration occurs when the asynchronous audio–visual stimuli are supposed to originate from the same event (i.e., having a common cause), and thence they are realigned^54^.

We found that the development of rapid recalibration emerged specifically for the audio–visual pairing, and not for the audio–tactile and visual–tactile pairings at any age. These results are consistent with previous studies in adults^23–25,30,31,55^, suggesting that short-term malleability is more pronounced for audio–visual than for audio–tactile or visual–tactile pairings. Note that in experimental settings, like those we used, the locations of the audio and visual stimuli were very close^5^, whereas the locations of the audio–tactile and visual–tactile stimuli differed^6,7^. This difference in locations may have reduced the likelihood of multisensory integration^9^ and therefore the necessity of rapid recalibration. However, the potential influence of the locations of multisensory stimuli on rapid recalibration has been ruled out in prior studies^23,24,55^.

The need for rapid recalibration may stem from the nature of processing of each cross-modal combination. In cross-modal simultaneity perception, the arrival time difference between each signal is determined by their physical transmission time to receptors and neural transduction time to the associated brain areas^56^. For the audio–visual pairing, because both stimuli are distal and the speed of light is much faster than sound, the signal arrival time difference is distance-dependent. To accommodate rapidly such variations of arrival time differences, the audio–visual system can modify the PSS according to the estimated distance of the source^57–59^ or, even more straightforwardly, according to the asynchrony of the most recent experience (i.e., rapid recalibration). For audio–tactile and visual–tactile pairings, the tactile stimulus is proximal on the body’s surface, and therefore the variability of the arrival time differences is small, caused mainly by the different neural transduction times when stimulating different locations on the body^23,31^. Thus, the fact that rapid recalibration develops mainly in audio–visual simultaneity perception but only mildly, or not at all, in audio–tactile or visual–tactile simultaneity perception appears to indicate that greater malleability is maintained for the audio–visual pairing because of the greater variation in daily experience.

Our findings also reveal that the relation between rapid recalibration and precision in multisensory simultaneity perception is not straightforward through development. Contrary to Noel et al.^35^, who demonstrated a decrease of rapid recalibration and the width of audio–visual simultaneity window (i.e., an improvement of precision) from childhood to adulthood, we did not observe a similar effect in any cross-modal pairings (see Fig. 2c). Further analyses within each age group showed that the relation depended on age and the type of cross-modal presentation. For audio–visual presentations, rapid recalibration and the precision of simultaneity perception is correlated by 11 years of age (see^25^, for the first report such correlation in adults). This correlation suggests that the wider window (i.e., lower precision) of audio–visual simultaneity perception in older children and adults may be attributable partly to higher susceptibility to the recent audio–visual asynchrony (i.e., a higher malleability), indicating a need to rethink the ecological meaning of the precision of audio–visual simultaneity perception. The correlation in the audio–visual pairing contrasted with the other cross-modal presentations, which demonstrated a general reduction in variance and a null correlation in later stages of development. Interestingly, we found a significant inverse relation in the youngest visual–tactile condition, which indicates a repulsive rapid recalibration when the window of simultaneity perception is wide. This trend may be spurious because of the higher variance in the young children’s behavioural measures, the unique performance of three children, or an overall preference for touch in visual-tactile judgments by 8 years of age^49^.

A limitation in the present study was that we examined only the relation between the development of simultaneity perception and rapid recalibration in the temporal domain, while the development of slow recalibration was not studied. Recent studies in the spatial domain suggest that audio–visual integration and rapid recalibration share a common neural substrate^36^, whereas the rapid and slow recalibrations tend to be dissociable in terms of their underlying mechanisms^60–63^ and developmental trajectories^4^. Inspired by the results in the spatial domain, it would be interesting to measure the development of slow recalibration together with rapid recalibration and the simultaneity window in order to gain a full picture in the temporal domain. Van der Burg et al.^24^ have developed a novel method to measure the rapid and slow recalibrations together, which is promising for future developmental studies.

In conclusion, we demonstrate that cross-modal rapid recalibration in the temporal domain emerged after multisensory simultaneity perception achieved adult precision for audio–visual presentations. In contrast, rapid recalibration in the audio–tactile and visual–tactile pairings did not develop at any age^31^. The uniqueness of rapid recalibration in the audio–visual system highlights its malleability to accommodate the stimulus arrival time difference depending on the distance of the source. Future investigations should examine whether maturation of audio–visual simultaneity perception is a prerequisite for the development of rapid recalibration; for example, it would be interesting to examine rapid recalibration in patients treated for congenital cataract because they show abnormal audio–visual simultaneity perception later in life^64^. The result will provide a contrast to the spared audio–visual integration and rapid recalibration in the spatial domain after early visual deprivation^65^.

## Methods

### The dataset

Cross-modal simultaneity judgment data were re-analyzed from three previous developmental studies^5–7^. All experimental protocols of these studies were approved by the McMaster Research Ethics Board. Methods in each study conformed to the Canadian Tri-Council Statement on Ethical Conduct of Research Involving Humans and the Declaration of Helsinki. All three studies recruited twenty participants for each age group that were balanced approximately by sex at birth. All three studies recruited independent groups of participants. The audio–visual experiment tested 5-, 7-, 9-, 11-year-olds, and adults. The age groups recruited in the audio–tactile and visual–tactile studies were similar, in that 7-, 9-, 11-year-olds, and adults were tested, with the exception that 13-year-olds were also tested in the visual–tactile study. Written consent was obtained from adult participants. For child participants, verbal assent was obtained from the child in conjunction with informed, written consent from their parents.

The procedures used to acquire each dataset are described in detail in the respective studies, that is, audio–visual^5^, audio–tactile^7^, and visual–tactile^6^. The following, however, provides a brief description. In the audio–visual experiments, participants fixated on the middle of a visual grey ring (~ 2° inner diameter), where a visual white disk (~ 2° diameter) was presented for about 17 ms on each presentation. The auditory stimulus was a 17 ms white noise burst presented from speakers on either side of the monitor. Audio–tactile stimuli were a 10 ms white noise burst presented through closed-ear headphones and a 10 ms dull tap delivered to the right index finger using a solenoid-based mechanical device aligned with the body midline. The same visual stimulus from the audio–visual experiment was used to record the visual–tactile simultaneity judgments, and the tactile stimulus was a 17-ms tap delivered to the right index finger, situated 20° below the visual stimulus and aligned with the participant’s body midline. In all three studies, participants reported orally if they perceived the individual onsets of the cross-modal stimuli to be synchronous or asynchronous. An experimenter seated beside participants recorded their responses and ensured that each participant adhered to the task requirements. A total of 13 stimulus onset asynchronies (SOAs) were tested for each cross-modal pairing: ± 1200, ± 800, ± 400, ± 300, ± 200, ± 100 or 0 ms. Negative SOAs indicate an auditory leading stimulus in audio–visual and audio–tactile experiments and a tactile leading stimulus in visual–tactile experiments. A total of 130 trials (10 trials per SOA) were collected for each 5-year-old participant, whereas a total of 260 trials (20 trials per SOA) were collected for older participants.

### Analysis

#### Data preparation

In our reanalysis of the data, we estimated the magnitude of rapid temporal recalibration for each participant’s dataset (and for each cross-modal condition) by first dividing the data into two bins based on the leading modality in the preceding trial. This resulted in two subsets for a specific age group and cross-modal condition, where modality A (audition in audio–visual and audio–tactile pairings, and touch in visual–tactile pairings) or modality B was the leading stimulus in the previous trial (see Fig. 1). A Gaussian distribution was fitted to these subsets and the amplitude, mean, and standard deviation were free parameters. The shift in the PSS was computed by subtracting the mean of the B-leading subset from the mean of the A-leading subset (i.e., PSS_A_–PSS_B_). Following earlier studies, we label A-leading SOAs as negative and B-leading as positive. Using this convention, rapid temporal recalibration exhibits a positive relationship with SOA^25^. That is, if modality A led on the previous trial, then the current trial PSS is shifted toward negative SOAs, and if modality B led on the previous trial, then the current trial PSS is shifted toward positive SOAs.

To ensure that the estimated PSS shifts were representative of the participants’ responses, we selected age groups with the same number of trials and excluded individuals with poor Gaussian fits (*R*^2^ < 0.60) on either subset. This eliminated data from the 5-year-olds because of fewer trials. Because of poor Gaussian fits, data were excluded for one 7-year-old and one 9-year-old were excluded from the audio–visual condition. For the audio–tactile condition, data collected from seven 7-year-olds, and one 9-year-old were excluded from the analysis. Finally, for the visual–tactile condition, data from four 7-year-olds, one 9-year-old, and one 13-year-old were excluded.

#### Main analyses

We conducted three analyses. The purpose of the first analysis was to determine the age at which rapid temporal recalibration becomes significant. PSS shifts of each condition were compared against a hypothesized PSS shift of 0 ms, which represented no rapid temporal recalibration. These comparisons were conducted using two-tailed, one-sample t-tests and bootstrap hypothesis tests. To conduct the bootstrap tests, 1500 bootstrap samples were generated for each pairwise comparison by resampling individual PSS shifts with replacement. The mean PSS shift of each iteration was then compared against 0 ms. Statistical significance was determined using the formula:

$$p = 2\min \left( {\frac{1}{B}\mathop \sum \limits_{j = 1}^{B} {\text {I}}( \overline{\Delta PSS}_{j} > C),\;\frac{1}{B}\mathop \sum \limits_{j = 1}^{B} {\text {I}}( \overline{\Delta PSS}_{j} < C)} \right)$$

which computes the two-tailed probability of having a mean PSS shift greater or less than the comparison value (denoted by C, here 0 ms). B is the total number of bootstrap samples and I(.) is the indicator function that returns a value of 1 when the PSS shift is either smaller or larger than 0 ms. By conducting both forms of statistical analyses, we could verify if our results were driven by individual data or if they were specific to the type of statistical test. For example, a positive *t*-test with a large effect size (e.g., *d* = 0.8) and a negative bootstrap test may indicate a biased sample.

The second analysis aimed to evaluate the differences in PSS shifts among three cross-modal pairings at a given age. Adult observers experience larger audio–visual rapid temporal recalibration effects than audio–tactile or visual–tactile pairings^23^, but it is unclear if a similar advantage can be observed across the different age groups. To investigate, PSS shifts of audio–tactile and visual–tactile pairings were evaluated against the PSS shifts of audio–visual pairings at the age groups common to all three datasets (i.e., 7-, 9- and 11-years-olds, and adults). As before, these comparisons were performed using two-tailed, independent-sample t-tests and bootstrap hypothesis tests. Similar bootstrap procedures were performed, with the exceptions that the test statistic was the difference in mean PSS shift and the comparison value was a mean difference of 0 ms.

In the final analysis, we investigated the correlation between the widths of the simultaneity window and PSS shifts across and within age groups. Broader audio–visual temporal simultaneity windows have been linked to larger PSS shifts in adults^25^; however, it is unclear if this correlation applies to younger age groups and other cross-modal presentations. Studies have shown that younger children have a wider window in which they make cross-modal simultaneity judgments^5–7,35^, but the wider windows might result from factors independent of the larger PSS shifts, such as needing more practice, inattention, or fatigue. One-way ANOVAs were conducted on each type of cross-modal presentation to examine the effect of age on the width of the simultaneity window or PSS shift. Separate linear regression analyses were also performed within each age group for each cross-modal pairing, and this allowed us to test how well the PSS shifts were correlated with the width of the simultaneity window. Similar bootstrap procedures were performed, and the same sample selections were used for PSS shifts and the width of the simultaneity window. In the within-age-group analysis, regression slopes were estimated for each of the bootstrapped samples, after which the probability of samples with slopes greater or less than 0 was computed. Main effects were assessed using bootstrapped *F* ratios, and we estimated the *p* value by computing the probability of obtaining ratios smaller or equal to 1 (equal variances).

## Data Availability

The datasets analysed during the current study are at the Open Science Framework repository, https://doi.org/10.17605/OSF.IO/NEZF3.
